# Diagnosis and neurosurgical treatment of glossopharyngeal neuralgia: clinical findings and 3-D visualization of neurovascular compression in 19 consecutive patients

**DOI:** 10.1007/s10194-011-0349-x

**Published:** 2011-05-13

**Authors:** C. Gaul, P. Hastreiter, A. Duncker, R. Naraghi

**Affiliations:** 1 Department of Neurology, University Duisburg-Essen, Hufelandstraße 55, 45147 Essen, Germany; 2 Department of Anaesthesiology and Operative Intensive Care, Martin-Luther-University Halle-Wittenberg, Halle, Germany; 3 Department of Neurosurgery, Armed Forces Hospital Ulm, Ulm, Germany

**Keywords:** Glossopharyngeal neuralgia, Neurovascular compression, Microvascular decompression

## Abstract

Glossopharyngeal neuralgia is a rare condition with neuralgic sharp pain in the pharyngeal and auricular region. Classical glossopharyngeal neuralgia is caused by neurovascular compression at the root entry zone of the nerve. Regarding the rare occurrence of glossopharyngeal neuralgia, we report clinical data and magnetic resonance imaging (MRI) findings in a case series of 19 patients, of whom 18 underwent surgery. Two patients additionally suffered from trigeminal neuralgia and three from additional symptomatic vagal nerve compression. In all patients, ipsilateral neurovascular compression syndrome of the IX cranial nerve could be shown by high-resolution MRI and image processing, which was confirmed intraoperatively. Additional neurovascular compression of the V cranial nerve was shown in patients suffering from trigeminal neuralgia. Vagal nerve neurovascular compression could be seen in all patients during surgery. Sixteen patients were completely pain free after surgery without need of anticonvulsant treatment. As a consequence of the operation, two patients suffered from transient cerebrospinal fluid hypersecretion as a reaction to Teflon implants. One patient suffered postoperatively from deep vein thrombosis and pulmonary embolism. Six patients showed transient cranial nerve dysfunctions (difficulties in swallowing, vocal cord paresis), but all recovered within 1 week. One patient complained of a gnawing and burning pain in the cervical area. Microvascular decompression is a second-line treatment after failure of standard medical treatment with high success in glossopharyngeal neuralgia. High-resolution MRI and 3D visualization of the brainstem and accompanying vessels as well as the cranial nerves is helpful in identifying neurovascular compression before microvascular decompression procedure.

## Introduction

Glossopharyngeal neuralgia is a rare entity with a reported incidence of 0.2–0.7/100,000/year. Epidemiological data are based on the extrapolation of few data from one center and recently from the analysis of a medical record database [1–4]. The frequency of glossopharyngeal neuralgia is underestimated. This is due to difficulties in clinical diagnosis, differentiation from trigeminal neuralgia and unawareness of the disease. Glossopharyngeal neuralgia shares several characteristics with trigeminal neuralgia: (a) paroxysmal attacks of brief electric shock-like stabbing pain, (b) trigger mechanisms, e.g., speaking, swallowing, eating, breathing, cold air and slight touch of the mouth and pharyngeal region, (c) initially good response to carbamazepine, and (d) association with neurovascular compression. Neurovascular compression as a source of irritation of cranial nerves was discussed first in 1929 by Dandy [5]. Jannetta’s operative work [12] [e.g., microvascular decompression (MVD)] provided evidence for this concept. The International Classification of Headache Disorders (ICHD) distinguishes between classical (ICHD-II 13.2.1) and symptomatic glossopharyngeal neuralgia (ICHD-II 13.2.2) [6]. However, based only on clinical findings a discrimination of both variants is not possible. “Idiopathic” cranial nerve neuralgias are mostly attributed to a neurovascular nerve compression syndrome at the root entry zone of the respective cranial nerve. The primary goal of diagnostic procedures is to rule out symptomatic glossopharyngeal neuralgia. Pain attacks in glossopharyngeal neuralgia are short (just for seconds), severe and stabbing in the pharyngeal region, including the tonsilar fossa, base of the tongue and below the angle of the jaw. The pain can also be felt in the areas innervated by the auricular and pharyngeal branches of the vagus nerve. Some authors propose distinguishing between a pharyngeal, otalgic and a vagal subtype of neuralgia and therefore suggest the term “vagoglossopharyngeal” neuralgia [7, 8].

A coincidence of trigeminal neuralgia and glossopharyngeal neuralgia in the same patient complicates the clinical diagnosis. However, pain attacks do not occur simultaneously in both areas [7]. Due to the rare occurrence of glossopharyngeal neuralgia, we report a case series of 19 patients to describe the clinical presentation, imaging findings and response to medical and neurosurgical treatment in detail.

## Methods

We report a series of 19 consecutive patients suffering from glossopharyngeal neuralgia referred for surgery due to unsatisfying response to previous medical treatment. The clinical appearance, pain localization, medical treatment and course of the disease were analyzed. The clinical symptoms were classified into pharyngeal, otalgic and vagal manifestation type of glossopharyngeal neuralgia. Data collection was done by retrospective analysis of the patients’ medical records. All patients were treated between 1994 and 2009 in the Department of Neurosurgery of the University of Erlangen-Nuremberg, Germany. Detailed prior history and clinical examination with special attention to cranial nerve function was done in all patients. All patients underwent high-resolution magnetic resonance imaging (MRI) and 3D visualization of the brainstem and accompanying vessels and cranial nerves in this region as described by Naraghi and Hastreiter [9–11]. Clinical data were correlated with MRI findings. The outcome and complications of 18 neurosurgically treated (MVD) patients were analyzed. For operative procedures, patients were placed in a semi-sitting position on the operating table; monitoring including precordial transthoracic echocardiography and somatosensory potentials was done, and a retrosigmoidal craniotomy was performed with extension to the foramen magnum. After incision of the dura, cerebrospinal fluid (CSF) was gathered from the cerebellopontine cistern (cisterna magna) and the cerebellar hemisphere. The inferior and biventral lobe was smoothly retracted to approach the lateral cerebellopontine cistern. The IX and X nerve were accessed at their exit zone from the brainstem and the conflicting vessel was identified. Dissection of the vessel was done according to the trigeminal nerve decompression suggested by Jannetta [12]. A Teflon interpositum was chosen to keep the vessel off [12].

All statistical analyses were performed with PASW Statistics 18.0.0. The demographic data of men and women were compared using *t* test analysis.

## Results

### Clinical picture and course of disease

Eleven men and eight women were investigated (Table 1). Mean age at onset of disease was 48.5 years [range 26–83, women (mean) 48.4 years, men (mean) 48.6 years]. Age at the time of MVD in the 18 operated patients was 54.5 years (mean). No significant difference was found between men and woman concerning either age of onset or age at the time of MVD. Twelve patients were affected on the left side and seven on the right side (1.7:1). Mean duration of disease before MVD was performed in 18 of the 19 patients was 6.5 (range 1–20) years. All 19 patients showed a pharyngeal pain manifestation. In addition, 12 patients showed symptoms of the otalgic type and three showed vagal-type symptoms (cough and raucousness accompanying the pain attacks, laryngeal pain attack localization). In two patients, additional trigeminal neuralgia was diagnosed. One of these had undergone MVD for trigeminal neuralgia on both sides previously (no. 17). Following the ICHD-II-criteria, classical glossopharyngeal neuralgia was diagnosed in 18 patients, one patient (no. 11) showed diminished sensibility in the palatine and pharyngeal area, but no other pathology than a neurovascular compression syndrome was diagnosed. In this patient, there was no other evidence of a symptomatic manifestation. In one patient, hearing loss and diminished sensibility in the trigeminal area was found, but these could be explained by previous thermocoagulation and a first MVD procedure in the past (no. 10). Patients with symptomatic glossopharyngeal neuralgia due to intracranial mass (tumor) or inflammation were excluded from our series. Medical treatment with anticonvulsants was used in all patients (mostly carbamazepine). All patients were treated with an adequate dose regime for several years. Failure to medical treatment was shown by all patients except one. By the time of evaluation for neurosurgical treatment, one patient (no. 1) was nearly free of symptoms under medical treatment with carbamazepine. Therefore, he did not undergo operation.

**Table 1:** Overview of epidemiologic data, case history, MRI findings, treatment and outcome of the patients

No.	Age/sex	Duration of disease (years)	Clinically affected cranial nerves	Main pain manifestation	Clinical findings	3D Visualzation: neurovascular contact	Course of the disease	Medical treatment previous to MVD	Operation performed	Course of the disease following MVD
1	68/m	10	Right IX, X	Pharyngeal, otalgic	CNE unremarkable	Compression of CN IX, X by PICA. VA ectasia and elongation on both sides	Under medical treatment, usually free of pain	CBZ	Not done	MVD not done
2	54/m	1	Left IX	Pharyngeal	CNE unremarkable, hypertension	Compression of CN IX, X by the VA and PICA	Initial improvement by medical treatment; during the course of disease loss of effectiveness of medical treatment	CBZ	MVD 2003	Transient hoarseness, normalization of hypertension, sustained pain-free status
3	45/f	2	Right IX, X	Pharyngeal, otalgic, additional constant pain in the left side of the face and the incisors	CNE unremarkable	Compression of the CN IX, X by PICA	Refractory to medical treatment	Not known	MVD 2004	Transient CSF hypersecretion, pulmonary embolism postoperatively, slight dropping of the palate to the right side, transient recurrent paresis. Pain free during the first week after surgery, afterward persistent dysesthesia of the cervical area on the right side
4	44/f	2	Left IX, X	Pharyngeal, later on additional vagal manifestation with cough and hoarseness during pain attacks	CNE unremarkable	Compression of the CN IX, X by PICA	During course of disease refractory to medical treatment	Gabapentin, pregabalin	MVD 2005	Transient CSF hypersecretion, sustained pain reduction, but not pain free. Transient recurrent paresis on the left
5	58/f	18	Left IX	Pharyngeal, otalgic	Hypesthesia of the right face, decrease of the right corneal reflex	Compression of the CN IX by PICA	During course of disease refractory to medical treatment	CBZ, gabapentin, pregabalin	MVD 1999 and 2005	Pain-free status for 2 years after the first MVD. Sustained pain-free status after the second MVD. Nonrecurring epileptic seizure after the second MVD
6	39/m	5	Left IX	Pharyngeal, otalgic	CNE unremarkable	Compression of the CN IX, X by PICA	Initially complete pain control by CBZ, later on refractory to medical treatment and increase of attack frequency	CBZ, gabapentin	MVD 2005	Sustained pain free, no complications
7	52/f	1	Left IX	Pharyngeal, otalgic	CNE unremarkable	Compression of the CN IX, X by PICA	Refractory to medical treatment, weight loss due to inability of regular food intake	Flupirtine, pregabalin	MVD 2005	Sustained pain-free status, no complications
8	57/m	3	Left V, IX	Pharyngeal, trigeminal	CNE unremarkable, hypertension	Compression of the CN IX, X by VA and compression of CN V by AICA	Initially trigeminal neuralgia, later on additional glossopharyngeal neuralgia refractory to medical treatment	Intolerability of CBZ, then on combination of topiramate, pregabalin, gabapentin, and amitriptyline	MVD 2007	Sustained pain-free status, transient recurrent paresis on the left side, dropping of the palate on the left
9	55/m	13	Left IX	Pharyngeal	CNE unremarkable	Compression of the CN IX, X by PICA	Initially good improvement by medical treatment, later on refractory to medical treatment	CBZ, gabapentin	MVD 2007	Sustained pain-free status, no complications
10	71/f	1	Left V, IX	Pharyngeal, otalgic	Hypesthesia in the face on the left side (caused by previous thermocoagulation of the ganglion Gasseri), vertigo, deafness on the left side	Compression of the CN IX, X by PICA	Initially left-sided trigeminal neuralgia, pain-free status for 5 years after thermocoagulation, MVD 19 years after onset of disease, followed by glossopharyngeal neuralgia. Second to MVD 20 years after onset of disease	CBZ	MVD 1992 and 1995	Sustained pain-free status, no complications
11	67/f	14	Right IX	Pharyngeal, otalgic	Pharyngeal and palatinal hypesthesia, hypertension	Compression of the CN IX, X by PICA	Initially good response to CBZ, later on refractory to medical treatment	CBZ	MVD 1996	Sustained pain-free status, no complications
12	63/f	7	Left IX	Pharyngeal, otalgic	CNE unremarkable	Compression of the CN IX, X by PICA	Initially good response to CBZ, later on refractory to medical treatment	CBZ	MVD 2000	Sustained pain-free status, no complications
13	58/m	6	Left IX	Pharyngeal	CNE unremarkable	Compression of the CN IX, X by PICA	Initially good response to medical treatment, pain-free status for 2 years, then refractory to medical treatment	CBZ, gabapentin, tramadol	MVD 2000	Sustained pain-free status, no complications
14	46/m	20	Left IX	Pharyngeal	CNE unremarkable	Compression of the CN IX, X by PICA	Initially good response to medical treatment, later on refractory to treatment	CBZ, gabapentin	MVD 2007	Sustained pain-free status, transient dysphagia
15	86/m	3	Right IX	Pharyngeal, otalgic, additional pain at the right throat	CNE unremarkable, hypertension	Compression of the CN IX, X by PICA	Refractory to medical treatment during the course of disease	CBZ, gabapentin, opioids	MVD 2009	Sustained pain-free status, no complications
16	55/m	1	Left IX	Pharyngeal, laryngeal	CNE unremarkable	Compression of the CN IX, X by PICA	Initially responsive to CBZ but with side effects, no satisfactory response to pregabalin	CBZ, pregabalin	MVD 2008	Sustained pain-free status, no complications
17	36/f	3	Right IX	Pharyngeal, laryngeal, otalgic	CNE unremarkable	Compression of the CN IX, X by PICA	2001 trigeminal neuralgia left sided, 2005 trigeminal neuralgia right sided, initially responsive to CBZ	CBZ	MVD 2001 (left V), MVD 2005 (right V), MVD 2008 (right IX and X)	Transient hoarseness, sustained pain-free status
18	49/m	5	Right IX	Pharyngeal, otalgic	CNE unremarkable	Compression of the CN IX, X by PICA	Initially responsive to CBZ, later on refractory to medical treatment	CBZ, gabapentin, pregabalin, amitriptyline, opioids	MVD 2007 not successful, MVD 2008	Sustained pain-free status, no complications
19	47/m	12	Right IX	Pharyngeal, otalgic	CNE unremarkable, hypertension	Compression of the CN IX, X by PICA and VA ectasia	Tonsillectomy was not successful as initial treatment, later on refractory to medical treatment	CBZ, gabapentin	MVD 2008	Sustained pain-free status, no complications

*CN* cranial nerve, *PICA* posterior inferior cerebelli artery, *VA* vertebral artery, *CBZ* carbamazepine, *MVD* microvascular decompression

### MRI findings

**Fig. 1:**
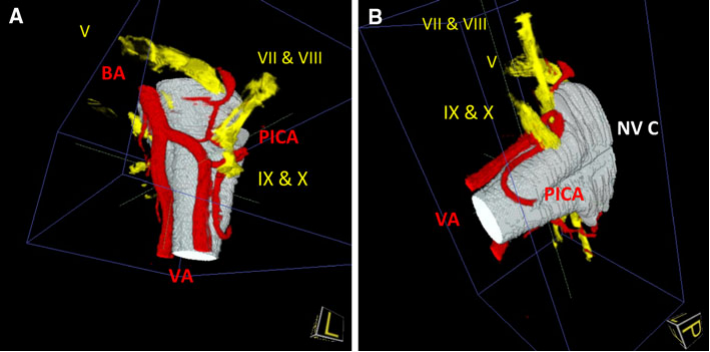
3D visualization of the neurovascular relationships in a case with left-sided glossopharyngeal neuralgia. With the presented method, we obtain a global overview of the neurovascular relations. We can move the picture in any direction and detect the presence of relevant vessels and cranial nerves and demonstrate the neurovascular compression at the root entry zone of the cranial nerves IX and X. The position as seen during microsurgery (compared to Fig. 2). *BA* basilar artery, *VA* vertebral artery, *PICA* posterior inferior cerebellar artery, *V* rigeminal nerve, *VII and VIII* facial and vestibulocochlear nerve, *IX* glossopharyngeal nerve, *X* vagus nerve, *NVC* neurovascular compression

**Fig. 2:**
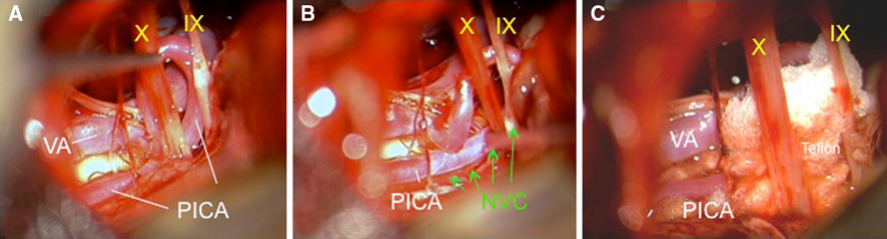
Intraoperative finding of the visualized case in** a**–**c**. The vertebral artery runs from caudal to rostral, while the (**a**) PICA runs in an upward loop close to the surface of the medulla and the root entry zone of the cranial nerves IX and X (**b**) inducing a neurovascular compression at this site. Adequate decompression was (**c**) achieved by insertion of Teflon. The intraoperative findings correspond very clearly to the results of the 3D visualization

All patients underwent high-resolution MRI, and the data were subjected to image processing and 3D visualization (illustrated in patient no 16) (Figs. 1, 2). Data were analyzed with 3D visualization revealing a neurovascular compression syndrome of the glossopharyngeal nerve in all patients, ipsilateral to the pain manifestation. In addition, MRI showed ipsilateral neurovascular compression of the vagal nerve in all patients. In two patients, additional ipsilateral neurovascular compression of the trigeminal nerve could be shown. Vessels, which were assumed to cause nerve compression, are listed for every patient (Table 1). Nerve compression by the posterior inferior cerebellar artery (PICA) was found in 15 patients, by the vertebral artery and PICA in three patients, and the vertebral artery plus the anterior inferior cerebellar artery (AICA) in another patient.

### Outcome of MVD

Of the 18 patients, 16 who underwent microsurgical decompression of the glossopharyngeal nerve were completely pain free after the procedure. They no longer needed any anticonvulsant or other pain medication. One patient (no. 4) improved substantially and medication could be reduced (she currently only takes gabapentin). One patient (no. 5) was pain free for 2 years, and then neuralgia returned. After a second MVD she was sustained pain free.

### Complications of MVD

Two patients (nos. 3 and 4) suffered from transient CSF hypersecretion as a reaction to the Teflon implant. CSF examination revealed lymphocytic pleocytosis. No causative bacteria could be found in CSF. Antibiotics were administered while CSF samples were taken repeatedly. Symptoms regressed within a few days. One patient (no. 3) suffered from postoperative deep vein thrombosis and pulmonary embolism, which prompted oral anticoagulation. Six patients showed transient cranial nerve dysfunctions with difficulties in swallowing or vocal cord paresis, but in all cases dysfunction resolved within 1 week. One patient (no. 3) complained of a gnawing and burning pain in the cervical area, but no signs of cranial nerve dysfunction were found on physical examination. Pre-existing disturbance in sensitivity in the trigeminal area of the face subsisted after surgical intervention. No patient suffered any hearing reduction as a result of surgery.

## Discussion

This case series focuses on symptomatology and response to medical and surgical treatment in patients suffering from glossopharyngeal neuralgia. Diagnosis should be considered if neuralgic pain occurs in the pharyngeal region or in the auditory canal. The coincidence of trigeminal and glossopharyngeal neuralgia that was found in two patients of our series (9:5%) is comparable to previous reports [3, 13, 14]. The coincidence of neuralgias of the V and the IX cranial nerves can be explained by the close anatomic relationship of the brainstem area, peripheral connections between both nerves and central convergence [7]. Neurovascular compression on the vagal nerve could be shown by MRI techniques in all of our 19 patients, but only three showed clinical symptoms corresponding to the vagal nerve. The infrequently clinically diagnosed coincidence of both neuralgias in contrast to the MRI findings may be explained by underreporting of vagal symptoms and subclinical involvement, such as asymptomatic bradycardia, which are not noticed by the patients themselves. Furthermore, it may be very difficult to decide whether syncopes in this population can be explained by vagal affection or the wide spectrum of alternative etiologies.

The presented data support MVD on the basis of image processing as a safe and promising second-line treatment after failure of standard medical treatment or unacceptable side effects of drug therapy. ICHD-II criteria (13.2.1) for classical glossopharyngeal neuralgia require an inconspicuous neurological examination (criteria D) [6]. More detailed clinical examination revealed subtle sensory deficit in some patients (nos. 5, 10, 11). Nevertheless, we did not see patients with any other lesion than neurovascular compression. This may be explained by a selection bias. Patients who were referred to neurosurgery and who underwent previous routine MRI, identifying symptomatic disease, may have been treated elsewhere.

In cases of glossopharyngeal neuralgia, MRI (including T2, T1, FLAIR) is suggested for the exclusion of symptomatic forms and for a first hint of neurovascular compression. MR-angiography (MRA) can show the anatomical relationship between cranial nerve and vessels (in particular arteries) [13]. In case of failure of medical therapy, or when unacceptable side effects occur, specialized MRI investigations including 3D constructive interference in steady state (CISS) are required. Using a specialized computer-based analysis, neurovascular compression in the root entry zone of the cranial nerve may be visualized as it was the case in all patients of our series (Fig. 1a, b) [9]. This finding is consistent with the findings of Akimoto [8] who showed that surgical findings in patients with trigeminal neuralgia correlate with MRI findings in 3D reconstruction from MRI imaging.

Of the 18 patients, 16 who underwent MVD became permanently pain free and the two remaining patients showed pain reduction. In former studies, pain-free rates in 76% of the patients and improvement in an additional 16% of patients were reported [15]. Long-term results of the intervention are good; after 10 years most patients remained pain free [14, 15]. As reported in literature, the PICA is the most frequent causative vessel, which compressed the root entry zone of the glossopharyngeal nerve in our series. This situation could be reliably visualized in all of the reported patients by high-resolution MRI and MRA, followed by image processing and 3D visualization. Accurate visualization is necessary before attempting operative therapy.

Risks of surgery consist of the common risk of anesthesiology, bleeding and infection. Special risks are hearing loss, hoarseness and difficulties in swallowing; these symptoms are temporary in the overwhelming majority of the patients. Teflon implant is a suspected cause of CSF hypersecretion, which results in postoperative headache. This was confirmed by a clinical study showing that lumbar puncture with drainage of 20–30 ml CSF results in sufficient headache relief [16]. To improve safety during the intervention, all patients underwent continuous intraoperative monitoring of the acoustic nerve and the lower cranial nerve EMG signals also in our series. We observed one life-threatening complication (pulmonary embolism in patient no. 3) and one patient with a non-recurring epileptic seizure (no. 5). All cranial nerve dysfunctions were transient (for details see Table 1). In a national registration of US hospitals 1,326 patients with trigeminal neuralgia, 237 with hemifacial spasm and 27 with glossopharyngeal neuralgia were reported. All patients were treated with MVD (Jannetta’s procedure) [17–19]. Mortality in this series was 0.3%; neurological deficit was reported in 1.7% of cases. The rate of adverse events depended on the frequency of the intervention in the hospital.

Of the 19 patients, 6 (32%) in our study suffered from additional arterial hypertension. Autoptic studies and clinical observations show weak evidence of a relationship between hypertension and neurovascular compression of the vagal and glossopharyngeal nerves, especially if left sided with compression of the rostral ventrolateral medulla oblongata [20–22]. Microvascular decompression improved arterial hypertension in some of these patients [20–22]. However, it has to be considered that hypertension is frequent in a cohort with a mean age of 54.5 years. Therefore, systematic follow-up with long-term blood pressure measurement is recommended in future studies.

## Conclusion

Patients suffering from uncommon facial pain syndromes including neuralgias of the cranial nerves should be treated in centers focusing on headaches and facial pain, if first-line medical treatment fails or diagnosis remains unclear. Interdisciplinary workup of cases opens the door to successful treatment even in difficult situations. Modern techniques of imaging such as high-resolution MRI and subsequent image processing with 3D visualization provide precise diagnosis of potential neurovascular compression of various cranial nerves (especially vagal, glossopharyngeal and trigeminal nerves). Microvascular decompression is a safe and successful treatment alternative in patients with failure or several side effects of medical treatment.
